# The Hypomethylating Agent 5-Azacitidine Potentiates the Effect of RAS and Sp1 Inhibitors in Neuroblastoma Cells

**DOI:** 10.32607/actanaturae.27558

**Published:** 2025

**Authors:** K. A. Ivanenko, A. V. Snezhkina, M. A. Zolotovskaia, P. V. Spirin, O. G. Leonova, V. I. Popenko, A. V. Kudryavtseva, A. A. Buzdin, V. S. Prassolov, T. D. Lebedev

**Affiliations:** Engelhardt Institute of Molecular Biology, Russian Academy of Sciences, Moscow, 119991 Russia; Moscow Institute of Physics and Technology, Dolgoprudny, 141701 Russia; Sechenov First Moscow State Medical University, Moscow, 119991 Russia; Center for Precision Genome Editing and Genetic Technologies for Biomedicine, Engelhardt Institute of Molecular Biology, Russian Academy of Sciences, Moscow, 119991 Russia; Shemyakin–Ovchinnikov Institute of Bioorganic Chemistry, Moscow, 117997 Russia; PathoBiology Group, European Organization for Research and Treatment of Cancer (EORTC), Brussels, 1200 Belgium

**Keywords:** pediatric malignant diseases, combination therapy, epigenetic regulators

## Abstract

Neuroblastoma is a malignant solid tumor caused by the transformation of neural crest cells. Neuroblastoma predominantly occurs in children and is associated with a poor prognosis. In this regard, the development of novel approaches to neuroblastoma treatment, including combination therapy, is relevant. DNA hypermethylation of neuroblastoma cells indicates that it is possible to use hypomethylating agents in a combination therapy of the disease. In order to identify effective combinations of antitumor drugs, we analyzed the transcriptomic changes that take place in neuroblastoma SH-SY5Y cells after treatment with the hypomethylating agent 5-azacitidine and then experimentally tested the effectiveness of these combinations. Mithramycin A and lonafarnib were the two drugs that, in combination with 5-azacitidine, appeared to exert a synergistic effect on SH-SY5Y cell death. These drugs inhibit the signaling pathway associated with the transcription factor Sp1 and RAS-MAPK signaling pathway, which are activated by 5-azacitidine. An analysis of the signaling pathways also revealed an activation of the signaling pathways associated with neuroblastoma cell differentiation, as well as apoptosis induction, as confirmed by multiplex and confocal microscopy. Hence, by analyzing the changes in the signaling pathways, the mechanisms of cell death and cell adaptation to hypomethylating agents can be understood, and this can be further used to develop novel therapeutic approaches to neuroblastoma therapy.

## INTRODUCTION

Neuroblastoma is an extracranial solid tumor that is the result of malignant transformation of neural crest cells during the formation of the sympathetic nervous system [1]. The five-year survival rate for children with high-risk neuroblastoma (50% of the cases) is approximately 60% [2]. The main treatment modalities for high-risk neuroblastoma include intensive chemotherapy, radiation therapy, autologous stem cell transplantation, and immunotherapy [3]. Targeted agents are under development: they would target disialoganglioside (GD2) [4], anaplastic lymphoma kinase (ALK) [5, 6, 7], E3 ubiquitin-protein ligase (MDM2) [8], and components of the signaling pathways such as the PI3K/Akt/mTOR, Fos/Jun, and RAS-MAPK pathways [9]. ALK inhibitors are already undergoing clinical trials for the treatment of patients with recurrent and refractory neuroblastoma [10]. The existing treatment approaches to such patients sometimes fail the test of effectiveness; therefore, combination therapies for neuroblastoma are now being pursued [11].

Alterations in DNA methylation are frequently observed in malignant cells of different origins, as well as hypermethylation of tumor suppressor promoters or global hypomethylation, in particular [12]. Two DNA methyltransferase inhibitors, 5-azacitidine (5-Aza) and its analog decitabine, have been approved for the treatment of myelodysplastic syndromes [13, 14]. 5-Aza is a hypomethylating agent and a synthetic analog of cytidine. Incorporating 5-Aza into DNA disrupts the activity of DNA methyltransferases, resulting in DNA hypomethylation and damage. The drug has been approved for the treatment of patients with acute myeloid leukemia and myelodysplastic syndromes [15].

Genomic DNA hypermethylation in neuroblastoma cells is associated with a poor prognosis [16]. 5-Aza was shown to induce the differentiation of neuroblastoma cells, reduce proliferation and colony formation, and to potentiate the cytotoxic effects of agents such as doxorubicin, cisplatin, and etoposide [17]. Decitabine has previously been tested in combination with doxorubicin; however, phase I clinical trials revealed the high toxicity associated with this combination [18]. Inhibitors of epigenetic regulators, such as histone deacetylase inhibitors, may exhibit synergism when used in combination with receptor tyrosine kinase inhibitors by upregulating their expression [19]. Additionally, 5-Aza can significantly affect the expression of the genes involved in oncogenesis through DNA demethylation. Therefore, it appears opportune to explore new therapeutic approaches that are based on the combination of 5-Aza with other antitumor agents.

This study analyzed the changes in gene expression and the activity of the signaling pathways in neuroblastoma SH-SY5Y cells exposed to 5-Aza in order to identify the most effective combinations of 5-Aza with various antitumor agents. The functional significance of alterations in the signaling pathway activity at the transcriptomic level was additionally examined by investigating intracellular processes using fluorescence microscopy and assessing the synergistic effects of 5-Aza and inhibitors of different signaling pathways. These findings can be used as a platform for developing novel therapeutic approaches to treat neuroblastomas susceptible to demethylating agents.

## MATERIALS AND METHODS

**Cell cultures and inhibitors**

Cell lines derived from human malignant tumors, including neuroblastoma SH-SY5Y, breast cancer SK-BR-3, renal cell carcinoma 786-O, cervical cancer SiHa, and ovarian cancer SK-OV-3 cells, were cultured in RPMI-1640 medium (Capricorn Scientific, Germany). Colorectal carcinoma HCT-116, lung adenocarcinoma H1299, glioblastoma LN-18, and rhabdomyosarcoma TE-671 cells were cultured in DMEM medium (Capricorn Scientific). All the cell lines were cultured at 37°C in a humidified atmosphere containing 5% CO_2_, supplemented with 10% fetal bovine serum (Gibco, USA), 1 mM sodium pyruvate (Gibco), 2 mM L-glutamine (Gibco), 100 U/mL penicillin, and 100 μg/mL streptomycin (Capricorn Scientific). The cells were passaged using phosphate-buffered saline and trypsin (ThermoFisher Scientific). The SH-SY5Y, H1299, LN-18, and TE-671 cells were provided by the Heinrich Pette Institute for Experimental Virology (Hamburg, Germany); the SK-BR-3 cells were obtained from the collection of the Institute of Cytology RAS (St. Petersburg, Russia); the remaining cell cultures came from the collection of the Engelhardt Institute of Molecular Biology RAS (Moscow, Russia). All the cell lines were regularly tested for mycoplasma contamination every two weeks using Hoechst-33342 DNA staining (Sigma- Aldrich, USA).

All the inhibitors used in this study were dissolved in dimethyl sulfoxide (DMSO); stock solutions were stored at –80°C (Table S1).

RNA for the transcriptome analysis was extracted from 1×10^6^ SH-SY5Y cells treated with 5 μM 5-Aza for 24 h. RNA extraction was performed using the phenol–chloroform method with the TRIzol reagent (Ambion), followed by treatment with DNase (Zymo Research, USA) and purification using RNA Clean & Concentrator-25 columns (Zymo Research), in accordance with the manufacturers’ protocols.

The quantity of extracted RNA was measured using the Qubit 4 fluorometer (Thermo Fisher Scientific). Total RNA integrity was assessed using an Agilent 2100 bioanalyzer (Agilent Technologies, USA). The RNA integrity number (RIN) for each sample was ≥ 8.

**RNA sequencing and transcriptome analysis**

A total of 1 μg of RNA was used to prepare each library. mRNA sequencing libraries were constructed using the TruSeq mRNA Library Prep Kit (Illumina, USA), in accordance with the manufacturer’s instructions. Various single-index adapters from the TruSeq RNA Single Index kits (Illumina) were ligated to each sample to facilitate multiplex sequencing. DNA fragments 250–300 bp long were selected using MagPure A4 XP magnetic beads (Magen Biotechnology, China). The cDNA libraries were then enriched by PCR and purified. Library quality was assessed using the Agilent 2100 Bioanalyzer. Equimolar amounts of the final libraries were pooled and sequenced on the NextSeq 2000 platform (Illumina) in the single-end mode with a sequenced read length of 101 bp. The sequencing data were analyzed using the STAR aligner software, version 2.7.4a [20], in the "GeneCounts" mode, with the Ensembl human transcriptome annotation (GRCh38 assembly version; GRCh38.89 transcript annotation). Raw RNA-seq expression values (in the ReadsPerGene format) were normalized according to the DESeq2 standard [21]. Pathway activation levels (PALs) were calculated for a total of 3024 pathways using an open-access collection of molecular pathways retrieved from the Oncobox pathway databank [22].

**Analysis of the activity of signaling** **pathways and CMAP analysis**

The CMAP algorithm was employed to identify similar or opposite effects [23]. This algorithm allows to compare the changes in gene expression induced in response to specific treatments and each perturbation out of the hundreds of thousands cataloged in the database. The CMAP algorithm indicates which perturbation affected gene expression in a way most similar or opposite to the analyzed treatment. In this study, we compared the expression levels of the 100 genes most significantly upregulated and the 100 genes most significantly downregulated under the experimental conditions. DMSO-treated SH-SY5Y cells were used as controls.

**Measurement of cell survival**

Cell survival was measured using the Cell Proliferation Assay XTT kit (11465015001, Roche, Sigma-Aldrich, USA) and an AbiCell Resazurin Cytotoxicity Assay Kit (CEL-04-30ML, Abisense, Russia).

SH-SY5Y cells (2,500 cells per well in a 96-well plate) were co-incubated with the compounds for six days; the growth medium was then removed, and resazurin or the XTT reagent was added to the cells. After 4 hours of incubation at 37°C in the presence of 5% CO_2_, the cell signaling level was measured using a Multiskan FC spectrophotometer by recording the difference in absorbance at 570 nm and 620 nm for resazurin, and at 450 nm and 605 nm for XTT. The changes in cell’s survival ability caused by the cocktail of drugs was calculated as the difference between the total effect of the drugs and the sum of their individual effects. The method used to measure the cell survival and to calculate area under the curve (AUC) was similar to that described previously [24]. In order to calculate the AUC, the area under the cell survival (%) vs. drug concentration curve was determined by dividing the diagram into trapezoids. The AUC values calculated for all the cell lines were used to obtain the mean AUC value, which was then used for normalization. The AUC value was normalized so that a AUC equal to 1 corresponded to the mean AUC values across all the cell lines.

**Cell counting on an automated microscope**

Cell counting was performed on an automated fluorescence microscope using the protocol for detecting cells that express the ERK-KTR H2B-mRuby reporter system, which was previously utilized in our laboratory [25]. Each experiment was performed in three replicates: four random imaging fields were selected in each well to count cells. The cells were imaged at four time points: 0, 24, 72, and 144 h. The images of the cells were obtained using Leica DMI8 fluorescence microscope (Leica, Germany); cell counts were completed using the Cellpose and CellProfiler software.

**Assessing the cell death mechanisms**

SH-SY5Y, cells were seeded into 96-well plates at a density of 2500 cells per well. Staining was performed 72 h after the addition of 5-Aza. The following dyes were used to visualize mitochondria, tubulin, lysosomes, FeII ions, caspases 3/7, nuclei, and DNA: TMRE (Lumiprobe, Russia), Tubulin TrackerTM Deep Red (Invitrogen, USA), LumiTracker® LysoGreen (Lumiprobe), HMRhoNox-M (Lumiprobe), NucView® 488 (Biotium, USA), Hoechst-33342, and 7-aminoactinomycin D (7-AAD) (BioinnLabs, Russia), respectively. Staining was carried out at 37°C in an atmos phere of 5% CO_2_. Imaging was performed using Leica DMI8 fluorescence microscope. Supplementary Table S2 summarizes the concentrations, staining durations, and imaging parameters.

Four images of the cells co-incubated with the compound at each concentration were recorded; the experiment was performed in two replicates. In the images, individual cells were identified using the Cellpose and CellProfiler software. The protocols for cell segmentation and fluorescence intensity measurements had been published previously [24]. The activities of the mitochondria, lysosomes, and FeII ions were quantified using the integral fluorescence intensity of each cell. The percentage of stained cells was determined in the CellProfiler software to analyze the caspase 3/7 activity and identify dead cells by 7-AAD staining.

**Confocal microscopy**

The cells were fixed with a 4% formaldehyde solution (Sigma-Aldrich, USA) in 0.1 M phosphate-buffered saline (PBS) for 15 min and subsequently blocked using a solution containing 1% bovine serum albumin (BSA) (PanEco, Russia), 22.52 mg/mL glycine (Sigma- Aldrich), and 1% Tween (Sigma-Aldrich) in PBS. Alexa Fluor® 647-conjugated antibodies (ab194322, Abcam, UK) were utilized to study the distribution of TRK receptor proteins in the cytoplasm. The cells were co-incubated with antibodies overnight at a 1 : 100 antibody dilution in a 1% BSA solution in PBS. Coverslips were placed cell-side down onto glass slides containing 8 μL of the Slowfade gold medium (Invitrogen, USA) with 1 μg/mL DAPI (Sigma- Aldrich), and sealed with nail polish. Nuclei were visualized using DAPI staining. The data were obtained by confocal microscopy using Leica TCS SP5 laser scanning microscope (Leica) equipped with an HCX PLAPO CS 63×1.4 oil-immersion objective lens. The recorded confocal images (8-bit format) were analyzed in the LAS AF 4.0 software.

**Data analysis**

Statistical tests and data visualization were conducted using the GraphPad Prism 8.0, Python, and LAS AF software. The mean values and standard deviations (SD) or cell viability assessment were calculated using R and GraphPad Prism 8.0.

## RESULTS

**Fig. 1:**
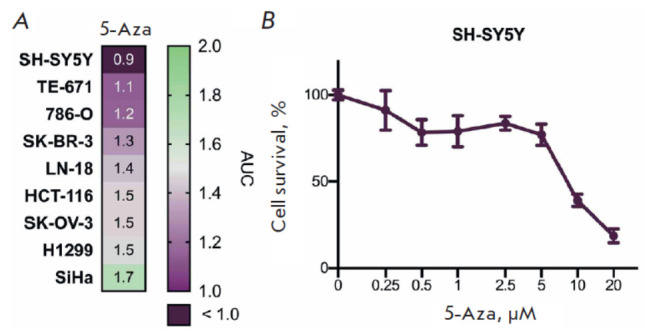
Toxicity assessment of 5-azacitidine for human neuroblastoma SH-SY5Y cells. (A) Sensitivity of malignant cells of different origins to 5-azacitidine (5-Aza) within 72 h. The cells were treated with the drug at concentrations of 0.25–20 μM; the figure shows the AUC (area under the curve) values. (B) Survival of neuroblastoma SH-SY5Y cells after 5-Aza treatment for 72 h. The graphs show the average value of three replicates and the standard deviation (SD). Cells incubated with dimethyl sulfoxide (DMSO) were used as controls

To assess the selectivity of 5-Aza toward neuroblastoma cells, we evaluated the effect of 5-Aza at concentrations ranging from 0.25 to 20 μM on various human cancer cell lines, including neuroblastoma SH-SY5Y, breast cancer SK-BR-3, renal cell carcinoma 786-O, cervical cancer SiHa, ovarian cancer SK-OV-3 colorectal carcinoma HCT-116, lung adenocarcinoma H1299, glioblastoma LN-18, and rhabdomyosarcoma TE-671 cells (Fig. 1A). The neuroblastoma SH-SY5Y cells were the ones most susceptible to 5-Aza (Fig. 1B).

Neuroblastoma SH-SY5Y cells were obtained by cloning a neuroblastoma SK-N-SH cell line [26]. SH-SY5Y is the cell line used in research most commonly: according to the data available at https://pubmed.ncbi.nlm.nih.gov/, the SH-SY5Y cell line was utilized in 13,789 publications, while the next most frequently used cell line, NMB, was mentioned in 5,338 publications. The SH-SY5Y cells harbor a mutation in the ALK gene (F1174L) [27] and are suitable for cell differentiation studies [28]. These cells exhibited the highest sensitivity to 5-Aza; so, further studies were performed using this cell culture.

**Fig. 2:**
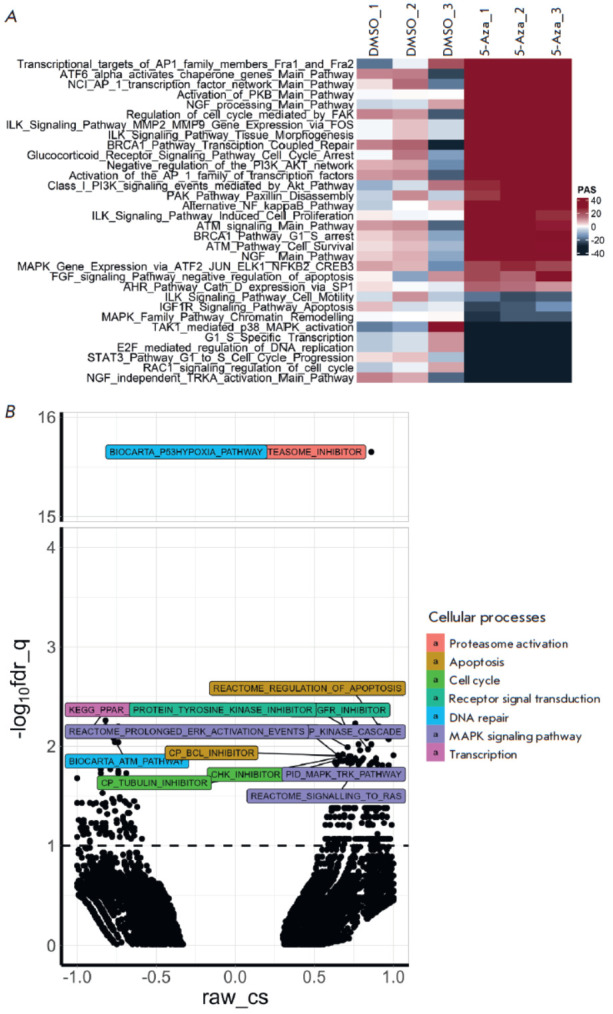
Changes in the signaling pathways in human neuroblastoma SH-SY5Y cells after treatment with 5-azacitidine (5-Aza). (A) Pathway activation strength (PAS) in SHSY5Y cells after treatment with 5 μM 5-Aza for 24 h according to the results of the Oncobox analysis [22]. The data are shown separately for each replicate. Pathway activation strength: the positive changes are shown in red; the negative changes are shown in black. The signaling pathways that may contribute to the progression of malignant tumors and incur statistically significant changes are shown. (B) Cellular processes that are altered in SH-SY5Y cells after treatment with 5 μM 5-Aza for 24 h according to the CMAP analysis. The dots indicate cellular processes from the CMAP analysis. Different colors indicate the classes of cellular processes with a reliable result according to the CMAP analysis. The results are shown as the inverse of the decimal logarithm of q-values after correction for multiple values (-log10fdr_q) and connectivity scores (raw_cs) and the degrees of similarity between differentially activated genes and the analyzed effect. Positive raw_cs values indicate identical changes in gene expression in response to treatment with 5-Aza and specified perturbations, while the negative values indicate opposite changes in gene expression in response to treatment with 5-Aza and specified perturbations

In order to identify which cellular processes are affected by 5-Aza in neuroblastoma cells, we conducted a transcriptome analysis of the cells treated with 5 μM 5-Aza for 24 h and compared the findings to those for the transcriptome of SH-SY5Y cells exposed to DMSO for 24 h. The transcriptome analysis data are reported as signaling pathway activities and gene expression profiles (Fig. 2A).

The most prominent positive changes in signaling activity were observed in the pathways associated with transcription factors AP1, ATF6, and Sp1, as well as protein kinase B (PKB) activation; nerve growth factor (NGF) processing; cell cycle arrest mediated by glucocorticoid receptors; the integrin-linked kinase (ILK) mediated signaling pathway, and the PI3K/Akt/ mTOR pathway (Fig. 2A). The most prominent negative changes were detected in the pathways associated with NGF-independent activation of receptor tropomyosin kinase A (TRKA), cell cycle, DNA replication involving the transcription factor E2F, the mitogen-activated protein kinase (MAPK) pathways, and apoptosis mediated by the insulin-like growth factor 1 receptor (IGF1R).

**Fig. 3:**
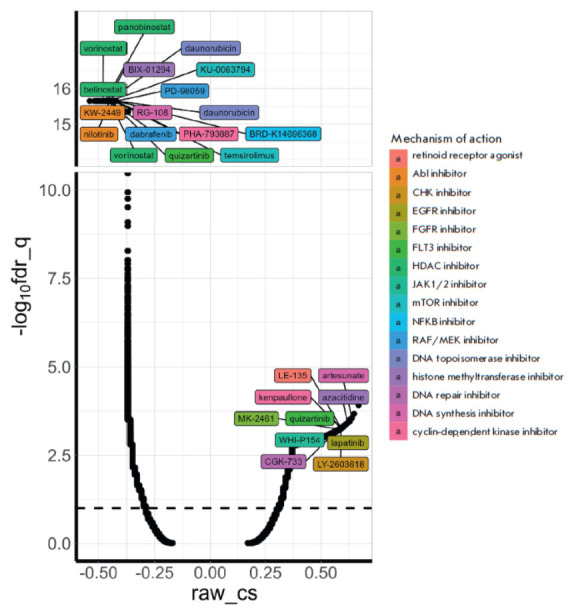
Identifying drugs with an effect similar to that of 5-azacitidine (5-Aza) on the gene expression of human neuroblastoma SH-SY5Y cells using CMAP. The cells were treated with 5 μM 5-Aza and co-incubated with the drug for 24 h. The dots indicate the effects of inhibitors, small hairpin RNAs, or overexpression of certain genes. Different colors indicate the classes of inhibitors. Drugs with a statistically significant maximal effect are shown. The results are presented as the inverse of the decimal logarithm of q-values after correction for multiple values (-log10fdr_q) and connectivity scores (raw_cs) and the degrees of similarity between differentially activated genes and the analyzed effect. Positive raw_cs values indicate identical changes in gene expression in response to treatment with 5-Aza and specified perturbations, while the negative values indicate opposite changes in gene expression in response to treatment with 5-Aza and specified perturbations

In order to determine what antitumor agents and cellular processes may exert a similar –or opposing – effect on the cellular transcriptomes, we conducted the CMAP analysis [23] (Tables S3 and S4; Fig. 2 and Fig. 3). Our analysis revealed significant alterations in the cellular processes in SH-SY5Y cells treated with 5-Aza (Fig. 2B). Such alterations were primarily related to the processes associated with the regulation of apoptosis and the cell cycle, proteasomal activity, receptor signaling, and the MAPK pathway (in particular, those associated with extracellular signal-regulated kinase (ERK) and TRK). Opposing changes were observed for the processes related to the response to DNA damage and transcription.

Inhibitors of the epidermal growth factor receptor (EGFR), the fibroblast growth factor receptor (FGFR), Janus kinase (JAK), cell cycle checkpoint kinase (CHK), and DNA repair affected gene expression in a manner similar to that for 5-Aza (Fig. 3). 5-Aza was found to be one of the compounds eliciting comparable effects, attesting to the validity of the observed transcriptomic changes. Opposing effects were induced by inhibitors of histone deacetylases, mTOR, topoisomerase, RAF serine/threonine kinase, tyrosine kinase Abl, and the transcription factor NF-kB. Inhibitors of cyclin-dependent kinases (CDKs), receptor tyrosine kinase (RTK) FLT3, and DNA synthesis had different effects on gene expression.

**Fig. 4:**
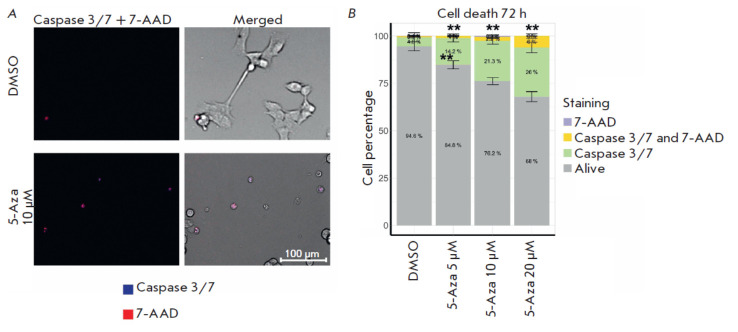
The contribution of apoptosis to the death of human neuroblastoma SH-SY5Y cells after treatment with 5-azacitidine (5-Aza) for 72 h. (A) Caspase 3/7 and 7-aminoactinomycin D (7-AAD) staining in SH-SY5Y cells after treatment with 10 μM 5-Aza. (B) Apoptotic cells (green and yellow) in a population of SH-SY5Y cells after treatment with 5–20 μM 5-Aza. Cells were imaged using an automated fluorescence microscope. Cells co-incubated with dimethyl sulfoxide (DMSO) were used as control. The analysis was performed based on an assessment of the fluorescence intensity of dyes in 350–2,500 cells; the standard deviation (SD) was estimated for the average values for eight images for each 5-Aza concentration. Statistical significance was determined vs. DMSO using the Mann–Whitney U test (**p ≤ 0.01)

**Fig. 5:**
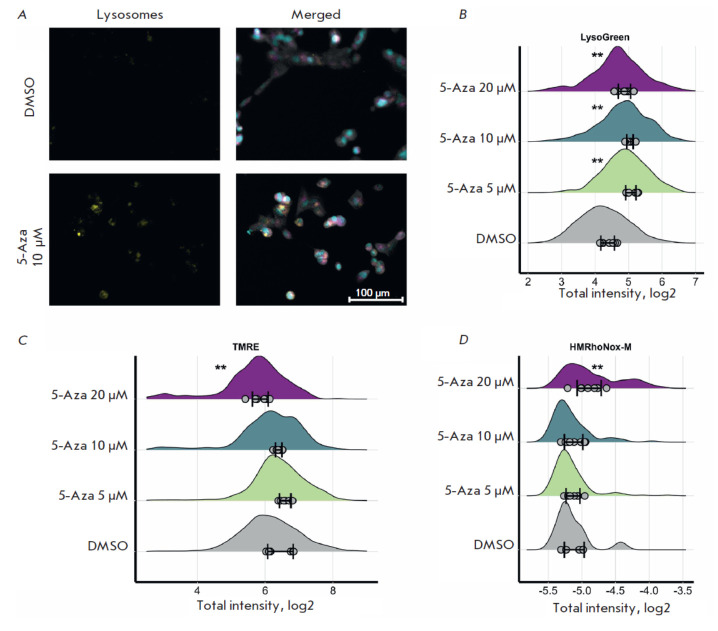
Changes in the lysosomal activity in human neuroblastoma SH-SY5Y cells after treatment with 5-azacitidine (5-Aza) for 72 h. (A) Lysosome staining in SH-SY5Y cells after treatment with 10 μM 5-Aza. Cells were imaged using a fluorescence microscope. Lysosomes are shown in yellow; nuclei, in blue; mitochondria, in magenta; tubulin, in gray. (B) Changes in the lysosomal activity in SH-SY5Y cells after treatment with 5–20 μM 5-Aza. (C) Changes in the mitochondrial activity in SH-SY5Y cells after treatment with 5–20 μM 5-Aza. (D) Changes in the FeII iron content in SH-SY5Y cells after treatment with 5–20 μM 5-Aza. Cells co-incubated with dimethyl sulfoxide (DMSO) were used as controls. The distributions of the integrated dye intensity in 350–2500 cells are shown; the average values for each of the eight images are shown with dots; the standard deviation (SD) for the average values for the images is also indicated. Statistical significance was determined vs. DMSO using the Mann–Whitney U test (**p ≤ 0.01)

We uncovered increased activities for seven apoptosis signaling pathways (Table S3). Since there exist several cell death mechanisms, we aimed to assess how 72 h treatment with 5-Aza would affect caspase 3/7, the mitochondria and lysosome activities, the Fe^2+^ content, and the number of dead SH-SY5Y cells. An up to 26% increase in the percentage of apoptotic cells was detected using fluorescent dyes (Fig. 4 A,B), thus attesting to the enhanced activity of the apoptosis signaling pathways. The lysosomal activity in SH-SY5Y cells was increased after 5-Aza treatment (Fig. 5 A,B). A slight decline in mitochondrial activity and increased Fe^2+^ levels were observed; however, these changes were minor and were likely to be related to cell death (Fig. 5 C,D).

**Fig. 6:**
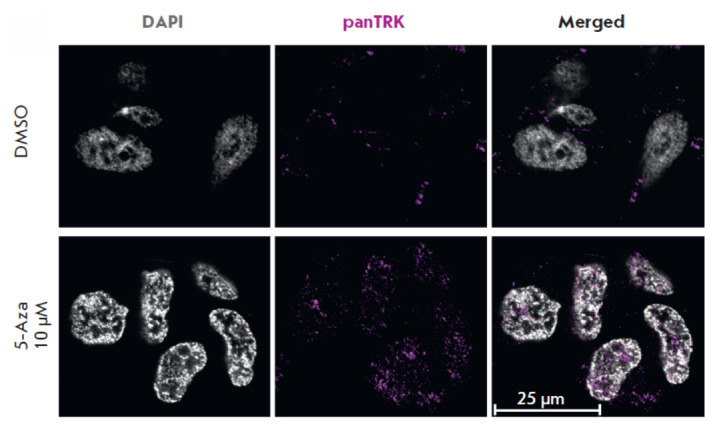
The distribution of TRK proteins in the cytoplasm of human neuroblastoma SHSY5Y cells after treatment with 10 μM 5-azacitidine (5- Aza) for 72 h. Cells co-incubated with dimethyl sulfoxide (DMSO) were used as controls. Cells were imaged by confocal microscopy using anti- TRK antibodies (Alexa647, magenta) and by staining the nuclei of fixed cells with DAPI (gray)

In vitro studies have demonstrated that NGF can inhibit the proliferation of neurogenic cancer cell lines and induce their differentiation [29]. Since 5-Aza affects the activity of the signaling pathways mediated by NGF and its receptor TRKA, we assessed the distribution of TRK receptors within the cytoplasm of SH-SY5Y cells treated with 10 μM 5-Aza for 72 h (Fig. 6).

The observed increase in the intensity of the staining of SH-SY5Y cells with anti-TRK antibodies can explain the enhanced activity of the NGF- and TRK-mediated signaling pathways at the gene expression level.

**Fig. 7:**
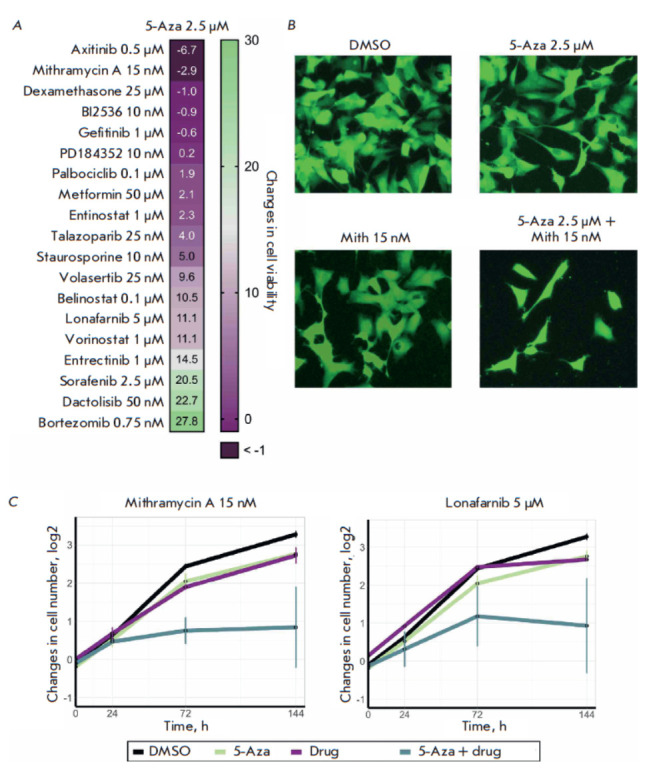
The effectiveness of combinations of 5-azacitidine (5-Aza) and antitumor drugs against human neuroblastoma SH-SY5Y cells. The cells were simultaneously treated with 2.5 μM 5-Aza and an antitumor drug (the drugs and their concentrations are shown in the figure) and co-incubated for 144 h. Cells co-incubated with dimethyl sulfoxide (DMSO) were used as controls. (A) The heatmap showing the synergistic effect of a combination of 5-Aza and inhibitors belonging to different classes for SH-SY5Y cells. (B) Images of SH-SY5Y cells expressing the ERK-KTR H2B-Ruby reporter system after treatment with a combination of 2.5 μM 5-Aza and 15 nM mithramycin A (Mith) for 144 h. Cells were imaged by fluorescence microscopy. (C) The diagrams of changes in the number of SH-SY5Y cells after simultaneous addition of 2.5 μM 5-Aza and 15 nM mithramycin A or 5 μM lonafarnib. The diagrams show the average value of three replicates and the standard deviation (SD)

Based on our findings (Tables S3 and S4; Fig. 2, and Fig. 3), we selected 18 drugs that should be further tested, in combination with 5-Aza. In particular, we chose a number of inhibitors targeting RTK, histone deacetylases, the MAPK pathway, the cell cycle, as well as proteasomes, glucocorticoid receptors, DNA synthesis, DNA damage repair, apoptosis inducer, and the activator of the p38 signaling pathway (Table 1).

**Table 1:** Selected drugs for assessing the effectiveness of their synergistic action on SH-SY5Y cells when used in combination with 5-azacitidine

Drug	Inhibitor class
Axitinib, entrectinib, gefitinib, sorafenib	Tyrosine kinase inhibitors
Belinostat, entinostat, vorinostat	Histone deacetylase inhibitors
Bortezomib	Proteasome inhibitor
Dexamethasone	Glucocorticoid receptor inhibitor, differentiation agent
Lonafarnib, PD184352	MAPK inhibitors
Mithramycin A	DNA synthesis inhibitor, inhibitor of Sp1 transcriptional activity
Metformin	Activator of the JNK/p38 MAPK pathway
BI2536, palbociclib, volasertib	Cell cycle inhibitors
Staurosporine	Apoptosis inducer, PKC inhibitor
Talazoparib	DNA damage response inhibitor

We simultaneously treated the cells with 2.5 μM 5-Aza and a second inhibitor at a pre-determined effective concentration that reduced cell viability by 20–50% within 72 h. The cells were subsequently incubated for another 144 h; cell viability was measured using a resazurin dye to assess the effectiveness of the drug combinations (Figure 7A).

The two most effective combinations – 5-Aza with axitinib, a multikinase inhibitor, and mithramycin A, a DNA synthesis inhibitor – were identified. However, since this cell proliferation assessment method shows changes in the cellular metabolic activity [24], we tested the viability of the cells treated with combinations of inhibitors and 5-Aza by counting the cells on an automated fluorescence microscope (Figure 7 B,C, S1). We observed that the findings differed from those obtained in the experiment using resazurin dye: the highest effectiveness was attributable to the combinations of 5-Aza with mithramycin A and lonafarnib, a small G protein inhibitor (RAS) (Figure 7C). Combinations of mithramycin A or lonafarnib with 5-Aza at concentrations that have no significant effect on cell proliferation led to substantial inhibition of SH-SY5Y cell growth and almost entirely stopped their proliferation.

## DISCUSSION

We analyzed the changes in the transcriptome of human neuroblastoma SH-SY5Y cells treated with 5-Aza in a search for potential drugs that could be used in combination with 5-Aza. The mechanisms through

5-Aza was found to affect the NGF-activated signaling pathways: it increases the intensity of cell staining using anti-TRK antibodies and alters the cell morphology. Earlier, it was demonstrated that 5-Aza induces the differentiation of neuroblastoma cells [30]. Among other factors, its action may have to do with the activation of cell differentiation. Some studies suggest that, in differentiated neuroblastoma cells, the absence of NGF induces apoptosis [31]. Differentiation induction by retinoic acid [32, 33] is extensively utilized to treat low-risk neuroblastoma and as maintenance therapy for the more aggressive forms of the disease [34]. A combination of 5-Aza and retinoic acid was shown to enhance the differentiation of neuroblastoma cells [35]. 5-Aza increases caspase 3/7 activity, which may be associated with cell differentiation and upregulation of the TRK receptor expression in the absence of NGF.

We observed an increased lysosomal activity in SH-SY5Y cells. 5-Aza has also been shown to induce autophagy in acute myeloid leukemia cells [36]. A hypothesis has been put forward that 5-Aza can trigger various neuroblastoma cell death pathways; however, further research is needed to verify this hypothesis. A transcriptome analysis of SH-SY5Y cells revealed alterations in the pathways linked with mitochondria and cell death; nonetheless, we have detected no significant changes in mitochondrial activity.

Mithramycin A is an antibiotic active against lung, esophageal [37], colorectal cancer [38], as well as leukemia cells [39]; however, this drug has been found to be highly toxic [40]. Lonafarnib has been tested in combination with ALK inhibitors in ALK-mutant neuroblastoma cells both in vitro and in vivo [41]. It has been demonstrated that both mithramycin A and lonafarnib can reduce DNA methylation levels [42, 43]. The enhanced effectivity of 5-Aza in SH-SY5Y cells when used in combination with these drugs can potentially be mediated by their effects on DNA methyltransferase 1 (DNMT1). Furthermore, neuroblastoma cells are strongly dependent on the activation of certain growth factor receptors [44, 45]. 5-Aza triggers several RTK-mediated signaling pathways, while lonafarnib can block signal transduction from RTK by inhibiting RAS [41].

## CONCLUSIONS

The analysis of the changes in the transcriptome of cells exposed to 5-Aza has identified drugs that exert a synergistic effect on neuroblastoma cell death and, in particular, the synergistic effect of a combination of 5-Aza and mithramycin A and lonafarnib against neuroblastoma SH-SY5Y cells. Further studies focusing on the effectiveness of drug combinations can pursue a more thorough analysis of the mechanism of the synergistic effect of these drugs and test the drug combinations in other neuroblastoma models.

## Abbreviations

5-Aza: 5-azacitidine
GD2: disialoganglioside
ALK: anaplastic lymphoma kinase
MDM2: murine double minute 2
DMSO: dimethyl sulfoxide
PKB: protein kinase B
NGF: nerve growth factor
ILK: integrin-linked kinase
TRK: tropomyosin receptor kinase
IGF1R: insulin-like growth factor 1
MAPK: mitogen-activated protein kinase
ERK: extracellular signal-regulated kinase
EGFR: epidermal growth factor receptor
FGFR: fibroblast growth factor receptor
JAK: Janus kinase
CHK: checkpoint kinase
mTOR: mammalian target of rapamycin
RAF: Rapidly Accelerated Fibrosarcoma, serine/threonine protein kinase
CDK: cyclin-dependent kinase
RTK: receptor tyrosine kinase
RAS: Rat Sarcoma, small G protein

## References

[1] Matthay K.K., Maris J.M., Schleiermacher G., Nakagawara A., Mackall C.L., Diller L., Weiss W.A. (2016). Nat. Rev. Dis. Prim..

[2] Irwin M.S., Naranjo A., Zhang F.F., Cohn S.L., London W.B., Gastier-Foster J.M., Ramirez N.C., Pfau R., Reshmi S., Wagner E. (2021). J. Clin. Oncol..

[3] Krystal J., Foster J.H. (2023). Children..

[4] Straathof K., Flutter B., Wallace R., Jain N., Loka T., Depani S., Wright G., Thomas S., Cheung G.W.-K., Gileadi T. (2020). Sci. Transl. Med..

[5] Foster J.H., Voss S.D., Hall D.C., Minard C.G., Balis F.M., Wilner K., Berg S.L., Fox E., Adamson P.C., Blaney S.M. (2021). Clin. Cancer Res..

[6] Fischer M., Moreno L., Ziegler D.S., Marshall L.V., Zwaan C.M., Irwin M.S., Casanova M., Sabado C., Wulff B., Stegert M. (2021). Lancet Oncol..

[7] Liu T., Merguerian M.D., Rowe S.P., Pratilas C.A., Chen A.R., Ladle B.H. (2021). Cold Spring Harb. Mol. Case Stud..

[8] Chen L., Pastorino F., Berry P., Bonner J., Kirk C., Wood K.M., Thomas H.D., Zhao Y., Daga A., Veal G.J. (2019). Int. J. Cancer..

[9] Geoerger B., Morland B., Jiménez I., Frappaz D., Pearson A.D.J., Vassal G., Maeda P., Kincaide J., Mueller U., Schlief S. (2021). Eur. J. Cancer..

[10] Zage P.E. (2018). Children..

[11] Pieniążek B., Cencelewicz K., Bździuch P., Młynarczyk Ł., Lejman M., Zawitkowska J., Derwich K. (2024). Int. J. Mol. Sci..

[12] Das P.M., Singal R. (2024). J. Clin. Oncol..

[13] Kaminskas E., Farrell A., Abraham S., Baird A., Hsieh L.-S., Lee S.-L., Leighton J.K., Patel H., Rahman A., Sridhara R. (2005). Clin. Cancer Res..

[14] Steensma D.P. (2009). Leuk. Res..

[15] Hollenbach P.W., Nguyen A.N., Brady H., Williams M., Ning Y., Richard N., Krushel L., Aukerman S.L., Heise C., MacBeth K.J. (2010). PLoS One..

[16] Gómez S., Castellano G., Mayol G., Suñol M., Queiros A., Bibikova M., Nazor K.L., Loring J.F., Lemos I., Rodríguez E. (2015). Epigenomics..

[17] Jubierre L., Jiménez C., Rovira E., Soriano A., Sábado C., Gros L., Llort A., Hladun R., Roma J., de Toledo J.S. (2018). Exp. Mol. Med..

[18] George R.E., Lahti J.M., Adamson P.C., Zhu K., Finkelstein D., Ingle A.M., Reid J.M., Krailo M., Neuberg D., Blaney S.M. (2010). Pediatr. Blood Cancer..

[19] Vagapova E., Kozlov M., Lebedev T., Ivanenko K., Leonova O., Popenko V., Spirin P., Kochetkov S., Prassolov V. (2021). Biomedicines..

[20] Dobin A., Davis C.A., Schlesinger F., Drenkow J., Zaleski C., Jha S., Batut P., Chaisson M., Gingeras T.R. (2013). Bioinformatics..

[21] Love M.I., Huber W., Anders S. (2014). Genome Biol..

[22] Zolotovskaia M.A., Tkachev V.S., Guryanova A.A., Simonov A.M., Raevskiy M.M., Efimov V.V., Wang Y., Sekacheva M.I., Garazha A.V., Borisov N.M. (2022). Comput. Struct. Biotechnol. J..

[23] Subramanian A., Narayan R., Corsello S.M., Peck D.D., Natoli T.E., Lu X., Gould J., Davis J.F., Tubelli A.A., Asiedu J.K. (2017). Cell..

[24] Mikheeva A., Bogomolov M., Gasca V., Sementsov M., Spirin P., Prassolov V., Lebedev T. (2024). Cell Death Discov..

[25] Lebedev T.D., Khabusheva E.R., Mareeva S.R., Ivanenko K.A., Morozov A.V., Spirin P.V., Rubtsov P.M., Snezhkina A.V., Kudryavtseva A.V., Sorokin M.I. (2022). J. Biol. Chem..

[26] Biedler J.L., Roffler-Tarlov S., Schachner M., Freedman L.S. (1978). Cancer Research.

[27] George R.E., Sanda T., Hanna M., Fröhling S., Luther W. 2nd., Zhang J., Ahn Y., Zhou W., London W.B., McGrady P. (2008). Nature.

[28] Kovalevich J., Langford D. (2013). Methods Mol. Biol..

[29] Aloe L., Rocco M.L., Balzamino B.O., Micera A. (2016). J. Exp. Clin. Cancer Res..

[30] Bartolucci S., Rossi M., Longo A., Rossi M., Estenoz M., Momparler R.L., Santoro B., Augusti-Tocco G. (1989). Cell Differ. Dev..

[31] Nakagawara A., Arima-Nakagawara M., Scavarda N.J., Azar C.G., Cantor A.B., Brodeur G.M. (1993). N. Engl. J. Med..

[32] Bayeva N., Coll E., Piskareva O. (2021). J. Pers. Med..

[33] Lebedev T.D., Vagapova E.R., Prassolov V.S. (2021). Acta Naturae..

[34] Makimoto A., Fujisaki H., Matsumoto K., Takahashi Y., Cho Y., Morikawa Y., Yuza Y., Tajiri T., Iehara T. (2024). Cancers (Basel)..

[35] Almeida V.R., Vieira I.A., Buendia M., Brunetto A.T., Gregianin L.J., Brunetto A.L., Klamt F., de Farias C.B., Abujamra A.L., Lopez P.L. da C. (2017). Mol. Neurobiol..

[36] Noronha N., Durette C., Cahuzac M., E Silva B., Courtois J., Humeau J., Sauvat A., Hardy M.-P., Vincent K., Laverdure J.-P. (2024). Leukemia..

[37] Zhang M., Mathur A., Zhang Y., Xi S., Atay S., Hong J.A., Datrice N., Upham T., Kemp C.D., Ripley R.T. (2012). Cancer Research.

[38] Quarni W., Dutta R., Green R., Katiri S., Patel B., Mohapatra S.S., Mohapatra S. (2019). Sci. Rep..

[39] Vagapova E.R., Lebedev T.D., Tikhonova A.D., Goikhman B.V., Ivanenko K.A., Spirin P.V., Prassolov V.S. (2020). Mol. Biol..

[40] Baum M. (1968). Br. J. Cancer..

[41] Pucci P., Lee L.C., Han M., Matthews J.D., Jahangiri L., Schlederer M., Manners E., Sorby-Adams A., Kaggie J., Trigg R.M. (2024). Nat. Commun..

[42] Lin R., Hsu C.-H., Wang Y.-C. (2007). Anticancer. Drugs..

[43] Chen T., Cai C., Wang L., Li S., Chen L. (2020). Front. Pharmacol..

[44] Lebedev T., Buzdin A., Khabusheva E., Spirin P., Suntsova M., Sorokin M., Popenko V., Rubtsov P., Prassolov V. (2022). Int. J. Mol. Sci..

[45] Lebedev T., Vagapova E., Spirin P., Rubtsov P., Astashkova O., Mikheeva A., Sorokin M., Vladimirova U., Suntsova M., Konovalov D. (2021). Oncogene..

